# New insights into the transcriptional regulation of benzoxazinoid biosynthesis in wheat

**DOI:** 10.1093/jxb/erac244

**Published:** 2022-09-12

**Authors:** Elia Stahl

**Affiliations:** Department of Plant Molecular Biology, University of Lausanne, 1015 Lausanne, Switzerland

**Keywords:** Benzoxazinoids, BXD, herbivory, plant–insect interactions, specialized metabolites, transcriptional regulation, transcription factors

## Abstract

This article comments on:

**Batyrshina ZS, Shavit R, Yaakov B, Bocobza S, Tzin V.** 2022. The transcription factor gene *TaMYB31* regulates the benzoxazinoid biosynthetic pathway in wheat. *Journal of Experimental Botany***73**, 5634–5649.

**Benzoxazinoids (BXDs) are abundant indole-derived specialized metabolites in several monocot crop species, such as wheat, maize, and rye. They function in plant immunity against herbivorous arthropods and fungal pathogens, but also as iron chelators, in metal tolerance, and as allelochemicals. Although BXD biosynthetic pathways have been studied extensively and are well described, information about the transcriptional regulation of BXD biosynthesis is scarce. In the current issue of *JXB*, Batyrshina *et al.* (2022) identified the transcription factor gene *TaMYB31* in the tetraploid wheat *Triticum turgidum* and verified its function as a component of BXD metabolism in the hexaploid wheat *Triticum aestivum*, where it regulates constitutive and stress-inducible BXD biosynthesis.**

Plants produce a vast array of specialized metabolites which regulate growth, development, reproduction, and the tolerance to abiotic and biotic stresses (De-la-Cruz Chacón *et al.*, 2013; Stahl *et al.*, 2018; Erb and Kliebenstein, 2020). BXDs belong to the best-studied specialized metabolites in monocot crop species, such as wheat, maize, and rye. They are constitutively produced as pre-formed defense compounds but their biosynthesis is additionally inducible by various pest attacks (Wouters *et al.*, 2016). BXDs are mainly described for their role in plant immunity against herbivorous arthropods. They possess a direct insecticidal activity, as their breakdown products inhibit insect digestive proteases (Feng *et al.*, 1992; Wouters *et al.*, 2016). Moreover, various BXDs function in immunity against microbial pathogens, as allelochemicals, as iron chelators, in plant–soil feedback mechanisms, and in metal tolerance (Poschenrieder *et al.*, 2005; Hu *et al.*, 2018; Niculaes *et al.*, 2018; Zhou *et al.*, 2018; Cadot *et al.*, 2021). BXDs are synthesized from indole-3-glycerol phosphate (IGP), which is converted to indole, a reaction catalyzed by the indole synthase BX1 (BENZOXAZINONELESS 1) (Fig. 1). Subsequently, four oxygen atoms are introduced, which form the BXD core structure 2,4-dihydroxy-1,4-benzoxazin-3-one (DIBOA), through the action of the four cytochrome P450 monooxygenases BX2–BX5. Glycosylation, hydroxylation, and methylation (BX6–BX14) transform DIBOA into different storage forms, which can be deglycosylated by β-glucosidases on demand. Although the enzymes contributing to BXD biosynthesis have been extensively studied in maize and wheat, the transcriptional regulation of *Bx* genes has not been comprehensively investigated. So far, just a few transcription factors were described in regulating BXD biosynthesis in maize and none was functionally determined in wheat (Gao *et al.*, 2019).

**Fig. 1.**
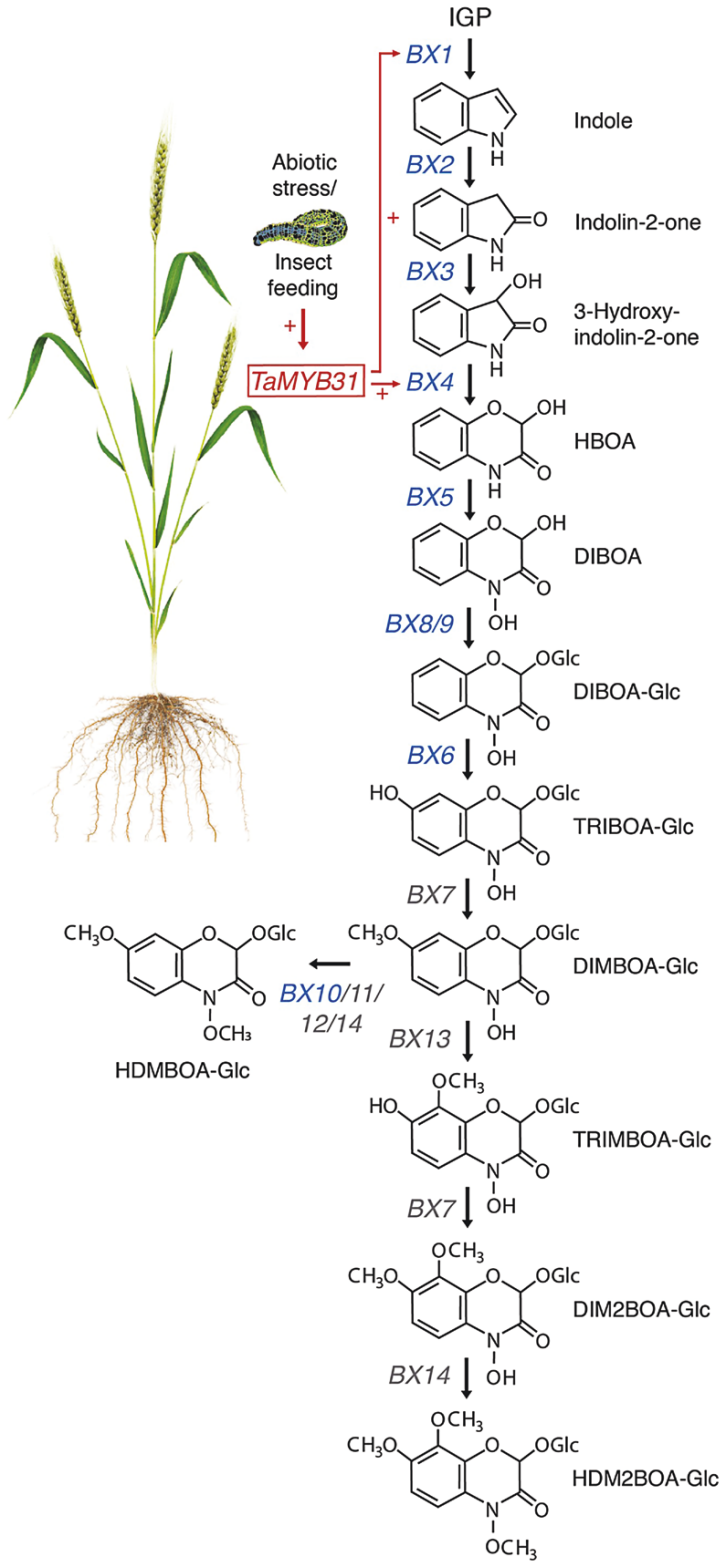
The involvement of TaMYB31 in benzoxazinoid biosynthesis in wheat. The biosynthetic pathway of benzoxazinoids (BXDs) in monocot crop species. Genes that were functionally characterized in maize and wheat are written in blue, genes that to date had just been identified in maize are written in gray. The first step of BXD synthesis is catalyzed by the indole synthase BX1, which converts indole-3-glycerol phosphate to indole. Afterwards, the four cytochrome P450 monooxygenases BX2–BX5 oxidize indole to the simplest BXD DIBOA, which is subsequently converted to various storage forms by a series of glycosylation, hydroxylation and methylation reactions (BX6–BX14). Glycosylated BXDs can be deglycosylated by β-glucosidases on demand. Batyrshina *et al.* (2022) demonstrated the involvement of *TaMYB31*, whose transcription is induced upon both abiotic and biotic stresses, in BXD biosynthesis in wheat by activating the promoters of the BXD biosynthesis genes *BX1* and *BX4*. Abbreviations: IGP, indole-3-glycerol phosphate; HBOA, 2-hydroxy-1;4-benzoxazin-3-one; DIBOA, 2;4-dihydroxy-1;4-benzoxazin-3-one; TRIBOA, 2;4;7-trihydroxy-2H-1;4-benzoxazin-3(4H)-one; DIMBOA, 2;4-dihydroxy-7-methoxy-1;4-benzoxazin-3-one; TRIMBOA, 2;4;7-trihydroxy-8-methoxy-1;4-benzoxazin-3-one; DIM2BOA, 2;4-dihydroxy-7;8-dimethoxy-1;4-benzoxazin-3-one; HDM2BOA, 2-hydroxy-4;7;8-trimethoxy-1;4-benzoxazin-3-one; HDMBOA, 2-hydroxy-4;7-dimethoxy-1;4-benzoxazin-3-one; Glc, glycosylated form of the corresponding compound.

## Identification of TaMYB31 as a component of BXD metabolism in wheat

The biosynthesis of BXDs is induced upon insect infestation (Glauser *et al.*, 2011; Wouters *et al.*, 2016). Batyrshina *et al.* (2022) exploited a recently published transcriptome dataset of tetraploid wheat (*Triticum turgidum*) to identify transcription factors up-regulated in response to insect herbivory (Shavit *et al.*, 2022). *In silico* prediction analysis indicated a potential interaction of TaMYB31, whose homoeologous genes were up-regulated in response to caterpillar and aphid infestation, with 25 *Bx* genes. To investigate the role of TaMYB31, the authors used the *Barley stripe mosaic virus*- (BSMV) based gene silencing system (VIGS) in seedlings of the hexaploid wheat *Triticum aestivum*. Silencing of *TaMYB31* resulted in decreased basal and herbivore-inducible levels of several BXDs, thus verifying a functional role for this transcription factor in BXD biosynthesis. Moreover, *TaMYB31*-silenced seedlings were more susceptible to insects from different feeding classes, including the bird cherry-oat aphid *Rhopalosiphum padi*, larvae of the Egyptian cotton worm *Spodoptera littoralis*, and the two-spotted spider mite *Tetranychos urticae*, confirming the broad role of BXDs in plant immunity against herbivores and the involvement of TaMYB31 in their biosynthesis.

TaMYB31 was previously reported to be involved in drought stress in wheat (Bi *et al.*, 2016). Batyrshina *et al.* (2022) therefore hypothesized that BXD metabolism could be additionally activated upon abiotic stresses and that *TaMYB31* could contribute to BXD biosynthesis under these conditions. Indeed, the authors reported a transcriptional activation of BXD biosynthesis upon drought and increased levels of various BXDs in response to drought.

To evaluate the interaction of TaMYB31 with promotors of *Bx* genes, the authors used a dual fluorescence transient expression system in *Nicotiana benthamiana*. TaMYB31 activated the promoters of *Bx1* and *Bx4*, which catalyze early steps in the BXD biosynthetic pathway (Fig. 1). Moreover, a network analysis of publicly available transcriptome datasets disclosed a co-expression of predicted *Bx* target genes with *TaMYB31* in response to biotic stresses such as pathogenic fungi, as well as upon abiotic stresses such as cold, drought, and heat. This analysis strengthens the hypothesis that TaMYB31 is a crucial regulatory component of BXD biosynthesis under both biotic and abiotic stress conditions.

## Missing links in the regulatory networks determining stress-inducible BXD metabolism

Due to the various functions of BXDs in agriculturally relevant crops, their biosynthetic pathways have been the subject of extensive research, and understanding the regulatory networks which underlie BXD metabolism holds great potential for the development of novel strategies for crop protection and pest management. Biosynthesis of BXDs has mainly been investigated in maize. Therefore, several enzymes involved in BXD synthesis have only been described in maize, but their orthologs in wheat have not been identified yet (Fig. 1) (Zhou *et al.*, 2018; Shavit *et al.*, 2022). Batyrshina *et al.* (2022) described here the first transcription factor regulating BXD metabolism in wheat. In addition to its contribution to basal BXD accumulation, TaMYB31 functions in stress-inducible BXD synthesis upon herbivore infestation (Fig. 1). Moreover, the authors demonstrated activation of BXD metabolism in response to drought and salt stress, and their analysis suggests an involvement of TaMYB31 in BXD synthesis in response to abiotic stresses. However, if and how BXDs contribute to abiotic stress tolerance in wheat are important and interesting aspects which deserve further investigation. Although the authors focused in the current study on the characterization of TaMYB31, their co-expression and *in silico* prediction analysis identified additional transcription factors (TaMYB29 and TaWRKY68) potentially functioning in BXD biosynthesis in wheat. Thus, they constitute promising candidates for additional regulation of BXD metabolism in wheat.

Albeit the accumulation of BXDs is observed in response to various pests, the molecular mechanisms which connect stress recognition with induction of BXD metabolism are currently not well understood. Recent findings indicate a role for jasmonic acid (JA) in BXD metabolism in wheat and maize, highlighting the regulatory role of JA in stress-inducible plant metabolic pathways (Richter *et al.*, 2021; Sue *et al.*, 2021). However, dependence of stress-inducible BXD accumulation on JA signaling has not been tested so far and is an additional intriguing aspect for further investigation. Moreover, the plant elicitor peptides ZmPEP1 and ZmPEP3 have been identified as signaling components in stress-inducible BXD biosynthesis in maize. ZmPEP1 and ZmPEP3 are induced in response to fungal infection and oral secretion of the generalist herbivore *Spodoptera exigua*. Upon PEP perception, maize plants activate a vast array of immune responses, including induction of BXD metabolism (Huffaker *et al*., 2011, 2013). A similar regulatory role has been recently described for SCOOP peptides in the model plant *Arabidopsis thaliana*, in which the perception of SCOOP peptides positively modulates the herbivore-inducible accumulation of indole glucosinolates (Stahl *et al.*, 2022). Therefore, how wheat endogenous peptides regulate BXD synthesis is an interesting question for future research.
